# Neutrophils and NETs in Pathophysiology and Treatment of Inflammatory Bowel Disease

**DOI:** 10.3390/ijms26157098

**Published:** 2025-07-23

**Authors:** Marina Ortega-Zapero, Raquel Gomez-Bris, Ines Pascual-Laguna, Angela Saez, Jose M. Gonzalez-Granado

**Affiliations:** 1 LamImSys Laboratory, Instituto de Investigación Sanitaria Hospital 12 de Octubre (imas12), 28041 Madrid, Spain; 2 Department of Immunology, Ophthalmology and ENT, School of Medicine, Universidad Complutense de Madrid (UCM), 28040 Madrid, Spain; 3 Department of Physiology, Faculty of Medicine, Universidad Autónoma de Madrid (UAM), 28029 Madrid, Spain; 4 Facultad de Ciencias Experimentales, Universidad Francisco de Vitoria (UFV), 28223 Pozuelo de Alarcón, Madrid, Spain; 5 Centro de Investigación Biomédica en Red de Enfermedades Cardiovasculares (CIBERCV), 28029 Madrid, Spain

**Keywords:** inflammatory bowel disease, ulcerative colitis, Crohn’s disease, neutrophil, NETs, tissue repair, reactive oxygen species, extracellular matrix remodeling, epithelial barrier, thrombus formation, therapy

## Abstract

Inflammatory Bowel Disease (IBD), which includes ulcerative colitis (UC) and Crohn’s disease (CD), results from dysregulated immune responses that drive chronic intestinal inflammation. Neutrophils, as key effectors of the innate immune system, contribute to IBD through multiple mechanisms, including the release of reactive oxygen species (ROS), pro-inflammatory cytokines, and neutrophil extracellular traps (NETs). NETs are web-like structures composed of DNA, histones, and associated proteins including proteolytic enzymes and antimicrobial peptides. NET formation is increased in IBD and has a context-dependent role; under controlled conditions, NETs support antimicrobial defense and tissue repair, whereas excessive or dysregulated NETosis contributes to epithelial injury, barrier disruption, microbial imbalance, and thrombotic risk. This review examines the roles of neutrophils and NETs in IBD. We summarize recent single-cell and spatial-omics studies that reveal extensive neutrophil heterogeneity in the inflamed gut. We then address the dual role of neutrophils in promoting tissue damage—through cytokine release, immune cell recruitment, ROS production, and NET formation—and in supporting microbial clearance and mucosal healing. We also analyze the molecular mechanisms regulating NETosis, as well as the pathways involved in NET degradation and clearance. Focus is given to the ways in which NETs disrupt the epithelial barrier, remodel the extracellular matrix, contribute to thrombosis, and influence the gut microbiota. Finally, we discuss emerging therapeutic strategies aimed at restoring NET homeostasis—such as PAD4 inhibitors, NADPH oxidase and ROS pathway modulators, and DNase I—while emphasizing the need to preserve antimicrobial host defenses. Understanding neutrophil heterogeneity and NET-related functions may facilitate the development of new therapies and biomarkers for IBD, requiring improved detection tools and integrated multi-omics and clinical data.

## 1. Inflammatory Bowel Disease: Clinical Features and Pathogenesis

Inflammatory bowel disease (IBD) encompasses two major idiopathic disorders—ulcerative colitis (UC) [1] and Crohn’s disease (CD) [2]—both characterized by chronic, relapsing inflammation of the gastrointestinal tract [3]. These disorders typically manifest early in life and affect both sexes, despite documented differences in phenotype and clinical course between males and females [4]. With over 6.8 million cases worldwide [5], the incidence and prevalence of IBD increased markedly from the mid-twentieth century onwards and continue to rise in newly industrialized regions. Although Europe and North America report the highest rates, these areas have experienced stabilization or decline in recent decades, while Asia, Africa, and South America face a growing disease burden [6,7]. Beyond digestive symptoms, IBD exerts a profound impact on education, employment, and family life [8].

Conventional therapeutic strategies, primarily aimed at modulating adaptive immune pathways, prove inadequate in 30–50% of patients due to insufficient response, adverse effects, or loss of efficacy over time [9]. The therapeutic landscape includes aminosalicylates, corticosteroids, and immunosuppressants—effective for short-term symptom relief but associated with hepatotoxicity, nephrotoxicity, and increased susceptibility to infections [10]; nutritional interventions that temporarily correct malnutrition but do not alter disease course [11]; and surgery, reserved for localized complications but often complicated by recurrence [12].

Lifestyle and dietary modifications are widely recommended, yet their efficacy varies significantly among individuals, limiting their generalizability and measurable impact [13]. Among novel modalities, microbiota-based approaches show therapeutic promise but currently lack consistent clinical validation, with trials yielding heterogeneous outcomes [14]. Likewise, stem cell therapy is still in the investigational phase, constrained by technical complexity, high cost, and limited clinical accessibility [15]. Despite these varied approaches, achieving sustained disease control and durable mucosal healing remains an elusive goal in clinical practice. Current interventions, while beneficial in certain contexts, frequently fail to induce durable remission or halt disease progression, underscoring the pressing need for more effective and targeted treatments.

Clinically, mucosal inflammation in IBD presents with recurrent abdominal pain, diarrhea, hematochezia, and weight loss, and is associated with extraintestinal complications and malignancy risk. Histologically, it is defined by infiltration of neutrophils and macrophages into the intestinal mucosa [3,16], innate immune cells that perpetuate inflammation through the release of pro-inflammatory cytokines, proteolytic enzymes, and reactive oxygen species (ROS), leading to epithelial injury and mucosal ulceration [17]. The absence of neutrophil infiltration in the lamina propria, together with resolution of neutrophil-induced damage in crypts and surface epithelium, is regarded as a key histological criterion for mucosal healing and remission [18]. In UC, inflammation remains confined to the mucosal and submucosal layers of the rectum and colon, where cryptitis and crypt abscesses constitute its defining histological lesions [19]. Patients typically endure episodes of watery to mucopurulent and bloody diarrhea, interspersed with asymptomatic intervals, and may develop extraintestinal manifestations of variable severity that further compromise quality of life [20]. By contrast, Crohn’s disease exhibits transmural inflammation, submucosal thickening, fissuring ulcerations, and non-caseating granulomas, affecting any segment of the gastrointestinal tract, but most frequently the terminal ileum, cecum, perianal region and colon. The clinical course of both disorders is defined by alternating periods of exacerbation and remission, with persistent inflammatory activity that not only impairs daily functioning but also increases morbidity and mortality risks [1,21].

### 1.1. Etiological Factors of IBD

Although the exact etiology of IBD remains incompletely understood, it is widely recognized that the condition arises from a complex interplay of genetic susceptibility, environmental factors, and immune dysregulation. Genetic predisposition plays a fundamental role, as evidenced by genome-wide association studies (GWAS) that have identified numerous susceptibility loci associated with both UC and CD [22,23]. Environmental exposures are thought to act as triggers in genetically predisposed individuals, initiating or exacerbating intestinal inflammation [24,25]. Infectious agents [26], dietary components [27], and disruptions in gut microbial composition [28] have all been implicated in disease pathogenesis. In addition, epithelial barrier dysfunction, dysbiosis of the intestinal microbiota, and aberrant immune responses contribute critically to the initiation and persistence of inflammation in IBD [29,30]. These factors collectively sustain chronic mucosal inflammation and tissue damage [31,32].

### 1.2. Immune Dysregulation and Genetic Susceptibility

IBD differs fundamentally from classical autoinflammatory diseases such as familial Mediterranean fever (FMF), Blau syndrome, and cryopyrin-associated periodic syndromes (CAPS), which are primarily caused by gain-of-function mutations in inflammasome-related genes and are characterized by excessive production of interleukin (IL)-1β and IL-18 [33]. In contrast, the pathogenesis of IBD is more commonly associated with loss-of-function mutations that compromise key innate immune functions. These defects often involve impaired epithelial barrier integrity and dysfunctional microbial sensing, leading to insufficient control of the gut microbiota and subsequent dysregulated activation of the adaptive immune system. This cascade contributes to chronic mucosal inflammation and progressive tissue damage [31,32,34]. While the roles of various innate immune components [31,34] and adaptive immune cells—particularly T lymphocytes [32]—in the pathogenesis of IBD are more clearly defined, the specific contributions of neutrophils have remained comparatively less defined. Underscoring the critical role of neutrophils in IBD, their infiltration into the intestinal mucosa represents a hallmark histological feature of active disease [35,36]. Moreover, several IBD susceptibility genes—including *NOD2*, *NCF4*, *LRRK2*, and *CARD9*—encode proteins integral to neutrophil-mediated microbial defense mechanisms [36]. The most widely used fecal biomarkers of disease activity are neutrophil-derived granule proteins, such as calprotectin (s100A8/9) that constitutes up to 60% of neutrophil cytosolic proteins [37,38,39,40,41], which reflect ongoing neutrophil activation and mucosal infiltration. Consistently, proteomic analyses have shown that levels of neutrophil-associated proteins markedly decline in patients achieving remission following treatment with various biologics [42]. These clinical and genetic observations suggest that neutrophils are not merely bystanders but active contributors to disease mechanisms. This review aims to provide a comprehensive overview of the role of neutrophils in IBD, encompassing both UC and Crohn’s disease CD. We cover both classical and recent findings, with particular attention to the functional heterogeneity of neutrophils and their dual role in intestinal inflammation. A special focus is placed on NETs, examining in detail their context-dependent effects that can either exacerbate or mitigate intestinal damage. This broader scope is intended to integrate past and emerging insights to guide future research and clinical perspectives. These insights may contribute to a better understanding of disease mechanisms and support the identification of new therapeutic targets.

## 2. Neutrophils in IBD Pathophysiology

Neutrophils, comprising nearly 70% of circulating leukocytes, are the most abundant immune cells in human blood and serve as a frontline component of the innate immune system, providing rapid defense against invading microbial pathogens [43]. In steady-state conditions, they patrol the vasculature, contributing not only to immune surveillance but also to various physiological processes including immune cell recruitment, angiogenesis, coagulation, and tissue regeneration [44]. Neutrophils play a paradoxical role in IBD, mediating both tissue damage [45] and host defense [46]. While their recruitment is critical for pathogen clearance and mucosal barrier maintenance, excessive activation leads to epithelial injury through the overproduction of ROS and proteolytic enzymes, which disrupt the intestinal mucosal barrier [47,48]. Persistent neutrophil infiltration further exacerbates inflammation by recruiting additional immune cells and activating redox-sensitive pathways, perpetuating tissue damage [47,48]. Conversely, neutrophils also contribute to inflammation resolution through mechanisms such as the secretion of pro-resolving mediators, highlighting their context-dependent regulatory roles [46,49,50].

### 2.1. Neutrophil Recruitment and Functional Diversity in IBD

Their development begins in the bone marrow, where progenitor cells undergo sequential differentiation into promyelocytes and subsequently mature into fully functional neutrophils [51]. These mature cells typically remain in a resting state within the peripheral circulation until activated [52]. Upon sensing infection or tissue injury, endothelial cells mediate neutrophil arrest and transendothelial migration, guiding these cells from the circulation into the inflamed mucosa [51]. Once within the intestinal lamina propria, neutrophils navigate toward sites of epithelial damage in response to a complex chemotactic milieu: pro-inflammatory cytokines such as interleukin-8 (IL-8), IL-6 and IL-33; chemokines including CXCL5, CXCL7, CXCL10 and CCL20; lipid mediators like leukotriene B4 (LTB4) and hepoxilin A3 (HXA3); and matrix metalloproteinases, notably MMP-3 and MMP-7. Together, these soluble factors establish precise spatial and temporal gradients that coordinate neutrophil recruitment and activation at loci of intestinal inflammation [53]. Interestingly, this transepithelial migration of neutrophils is associated with aggravated disease severity and crypt architecture disruption in IBD and experimental colitis models [53]. Genetic susceptibility in IBD involves key regulators of neutrophil trafficking, such as CXCL5, CCL20, MMP9, HCK, LSP1, and HGFAC, which influence recruitment and migration dynamics [53].

For many years, neutrophils were regarded as a uniform, short lived population primarily devoted to microbial killing. However, over the past years, accumulating evidence has revealed that neutrophils can adopt diverse phenotypes and functions under both homeostatic and pathological conditions [54]. Neutrophil heterogeneity has been uncovered through diverse and complementary methodologies. Initial insights came from density gradient centrifugation, which distinguished normal-density neutrophils (NDNs) from low-density neutrophils (LDNs)—a subset displaying either pro- or anti-inflammatory functions. In the context of cancer, further characterization has identified tumor-associated neutrophils with distinct N1 (anti-tumorigenic) and N2 (pro-tumorigenic) phenotypes [54,55]. Subsequent flow cytometric analyses have subdivided neutrophils according to surface markers—including CD177, OLFM4, CD10, CD33, CD11b/c, CD66b, CD63, CD62L, CD54, and CXCR4 [49,54,56,57,58]—with CD177^+^ cells proposed to confer protection in IBD [59]. It remains unclear, however, whether these populations represent stable neutrophil subsets or transient activation or maturation states induced by local tissue cues—including reverse-transmigrated cells returning from inflamed sites to the circulation [60]. Unbiased single-cell RNA sequencing has delineated maturation-dependent transcriptional states in both murine and human neutrophils, including an interferon-inducible terminal subset, underscoring the influence of environmental signals on neutrophil identity [61,62,63]. Moreover, single-cell transcriptomics combined with spatial imaging of colonic lesions in UC and CD patients identified neutrophils among the most transcriptionally diverse cell types, comparable to macrophages [64]. In these inflamed tissues, neutrophils localized to crypt abscesses and ulcerated regions exhibited upregulated CD62L, CD193, and CD69 relative to their peripheral counterparts, reflecting distinct activation states [64]. Three intestinal neutrophil states were defined: N1 and N3, which resembled peripheral neutrophils, and N2 characterized by elevated expression of genes involved in tissue localization, notably *CXCR4*, which is associated with inflamed tissue. N3 displayed an interferon-inducible signature [64]. Moreover, local characterization of neutrophil transcriptional programs—using murine and human single-cell RNA sequencing, combined with human spatial transcriptomics—has revealed distinct functional identities between lamina propria neutrophils and those associated with the epithelium in inflamed colonic tissue. These findings were further supported by real-time intravital imaging in murine models. Lamina propria neutrophils exhibited transcriptional profiles supporting enhanced motility and microbial uptake, whereas epithelium-associated neutrophils were enriched in hyperactivated or pro-apoptotic states, marked by elevated ROS levels and increased TNFα production [65]. These observations highlight the dynamic specialization of neutrophils in IBD and underscore the necessity for broader profiling to fully capture their functional diversity [64].

### 2.2. Inflammatory Mediators Derived from Neutrophils

Within the intestinal mucosa, neutrophils deploy key antimicrobial strategies—phagocytosis, neutrophil extracellular traps (NET) formation, and degranulation—to control and eliminate invading pathogens [46,49,50]. However, these same effector mechanisms can exacerbate tissue injury by releasing ROS, cytotoxic granule components—myeloperoxidase (MPO), defensins, neutrophil elastase (NE), proteases, and hydrolases—and by forming excessive NETs [66,67]. ROS compromise cellular membranes and activate redox-sensitive inflammatory pathways, perpetuating mucosal damage [68], and proteolytic enzymes, including MMPs and NE, degrade epithelial cell junctions, contributing to crypt deformation and abscess formation—hallmarks of IBD pathology [53]. Regarding epithelial injury during acute inflammation, neutrophil-derived junctional adhesion molecule-like protein (JAML) interacts with the epithelial tight junction protein coxsackie-adenovirus receptor (CAR), compromising barrier integrity and impairing mucosal healing [69]. Additionally, neutrophil-derived microparticles carrying surface-bound MPO disrupt intestinal epithelial repair by altering actin cytoskeleton dynamics and inhibiting cell proliferation [70]. Neutrophils secrete pro-inflammatory cytokines such as TNFα and IL-1β, which amplify immune dysregulation. Elevated IL-1β in UC promotes neutrophil activation, intestinal fibrosis, and barrier dysfunction via microRNA-200c-3p modulation [71]. IL-1β enhances granulocyte recruitment and drives autophagy-dependent NET formation decorated with bioactive IL-1β, regulated by REDD1 (DDIT4). REDD1 links oxidative stress and mTOR signaling to autophagy, potentially serving as a biomarker for IBD severity [72]. Regarding TNFα, it amplifies neutrophil recruitment, survival, and activity in IBD, primarily via NF-κB activation [73]. TNFα signaling through TNFR1 induces cell death via RIPK1 and caspase-3 activation [74], while promoting angiogenesis, macrophage activation, and T-cell survival through TRAF2/NF-κB [75]. TNFα disrupts epithelial integrity by compromising barrier function, inducing Paneth cell necroptosis [76], and stimulating MMP production by myofibroblasts [77]. Collectively, TNFα sustains a pro-inflammatory loop, driving tissue damage and chronicity in IBD.

## 3. Neutrophil Extracellular Traps (NETs)

Neutrophils undergo a unique form of cell death involving the release of chromatin into the extracellular space [78]. This process, termed NETosis, leads to the formation of NETs and is mechanistically distinct from other regulated cell death pathways such as apoptosis, necroptosis, and pyroptosis [79]. These DNA-based scaffolds serve not only to trap invading pathogens but also to enhance their elimination by the immune system [80]. Originally recognized for their antimicrobial function, NETs are now acknowledged as critical contributors to pathological inflammation, tissue injury [81], regulated cell death pathways [82], and tissue remodeling [83], thereby linking innate immunity to the development and progression of inflammatory disorders.

**Figure 1:**
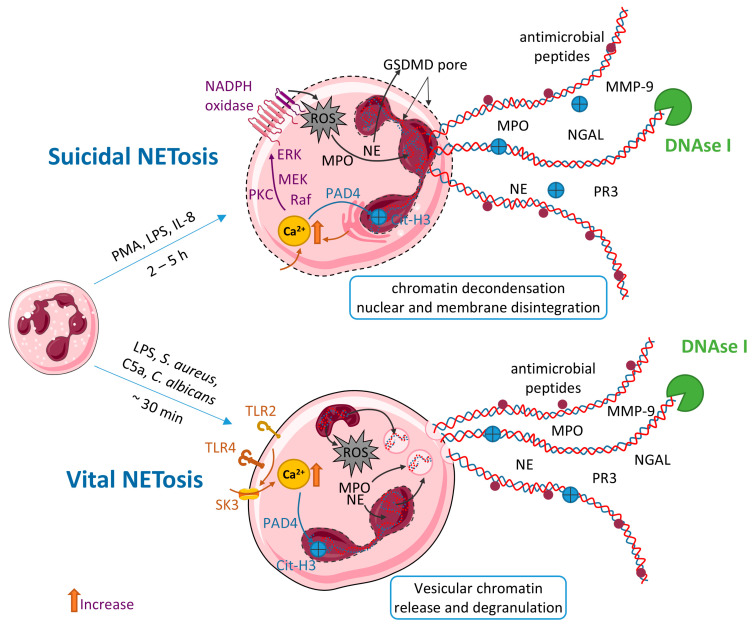
Mechanism of neutrophil extracellular trap (NET) formation. Neutrophil extracellular traps (NETs) consist of decondensed chromatin (DNA and citrullinated histones, Cit-H3) decorated with neutrophil granule proteins–including neutrophil elastase (NE), myeloperoxidase (MPO), neutrophil gelatinase-associated lipocalin (NGAL), proteinase 3 (PR3), and matrix metalloproteinase-9 (MMP-9)–together with antimicrobial peptides and PAD4. The NET scaffold contains gasdermin D (GSDMD) pores that facilitate chromatin release. NETosis proceeds via two principal pathways. In suicidal (NOX-dependent) NETosis, activation by stimuli such as phorbol esters (PMA), cytokines, or pathogens triggers a PKC–Raf–MEK–ERK signaling cascade, leading to NADPH oxidase activation, reactive oxygen species (ROS) production, histone citrullination by PAD4, nuclear envelope rupture, and, finally, cell lysis. In vital (NOX-independent) NETosis, stimulation of Toll-like receptors (TLR2/4) or SK3 calcium channels causes a Ca^2+^ influx and vesicular export of chromatin without immediate neutrophil death, preserving cell integrity. In both cases, NETs are ultimately degraded by extracellular DNases (primarily DNase I) that dismantle the chromatin mesh. Part of the figure was generated using images from Servier Medical Art.

NETs consist of a fibrous matrix primarily built from decondensed chromatin, including DNA and histones (H1, H2A, H2B, H3, and H4), which is interlaced with a variety of cytoplasmic and granular proteins [78,84]. Key granule-associated components of NETs include NE [85], MPO [86], neutrophil gelatinase-associated lipocalin (NGAL, also known as lipocalin-2, LCN2) [87], peptidylarginine deiminase 4 (PAD4) [88], proteinase 3 (PR3) [89], cathepsin G and MMP-9, lactoferrin, among others [78,90,91] shown in Figure 1. Each of these components plays a distinct role in shaping the functional and pathological properties of NETs. NE degrades elastin, a major component of the extracellular matrix, facilitating tissue remodeling and potentially contributing to tissue injury [92]. PAD4 is essential for NET formation through citrullination of histones, a modification that promotes chromatin decondensation [93,94]. MPO, in conjunction with hydrogen peroxide, generates ROS, enhancing both pathogen killing and collateral tissue damage [95]. PR3 amplifies tissue injury via proteolytic cleavage of structural proteins [96]. Moreover, NETs are enriched in antimicrobial peptides such as defensins, cathelicidins (cathepsin G), and lectins, which further contribute to pathogen neutralization [97]. Although the overall proteomic profile of NETs is relatively conserved, its composition may vary depending on the specific activating stimulus. This dynamic nature allows NETs to interact with other components of the immune system, shaping the inflammatory environment and influencing the pathogenesis of immune-mediated diseases, including CD [98].

Two principal modes of NET formation have been described: suicidal (or lytic) NETosis and vital (or non-lytic) NETosis Figure 1.

Suicidal NETosis is characterized by cell spreading, loss of nucleolar integrity, rupture of the nuclear envelope, breakdown of the plasma membrane and profound chromatin decondensation—ultimately culminating in neutrophil lysis. A variety of stimuli can induce this response, notably bacteria, fungi, pro-inflammatory cytokines, LPS, and especially PMA, which is recognized as the most potent NETosis trigger [99,100]. The stimulation of receptors elevates cytosolic Ca^2+^ concentrations [101]. This rise in Ca^2+^ activates protein kinase C (PKC), while PMA concurrently stimulates the RAF–MEK–ERK cascade. Together, PKC and RAF–MEK–ERK signaling drive the assembly and phosphorylation of the NADPH oxidase (NOX) complex, resulting in ROS production [99,102]. High intracellular Ca^2+^ also activates PAD4, which converts histone arginines to citrulline, weakening histone–DNA interactions and promoting chromatin relaxation [103,104]. Concurrently, ROS facilitate translocation of MPO and NE from azurophilic granules into the nucleus, where they contribute to histone cleavage and further chromatin decondensation [105] and modulate cytoskeletal dynamics [106]. Following nuclear envelope breakdown, decondensed chromatin mixes with granular contents in the cytosol and is expelled upon plasma membrane rupture, leading to cell death. In parallel, NE cleaves gasdermin D (GSDMD) into its pore-forming N-terminal fragment, which perforates the plasma membrane [107]. GSDMD activation supports both early nuclear membrane permeabilization and final membrane rupture [107]. Typically, the entire sequence from initial stimulus to neutrophil demise unfolds over 2–5 h.

In contrast to suicidal NETosis, NET release can occur independently of cell death and loss of plasma membrane integrity—a process termed vital NETosis [108]. In this mode, neutrophils extrude DNA-containing structures within approximately 30 min of activation, without undergoing lysis, thereby contributing to rapid antimicrobial defense [109]. Vital NET formation relies on a functional cytoskeleton [110] and is initiated by engagement of specific surface receptors. For example, *Staphylococcus aureus* and *Candida albicans* activate neutrophils via Toll-like receptor 2 (TLR2) and complement receptors, respectively, whereas LPS- or *Escherichia coli*–activated platelets signal through TLR4. Activation of these receptors opens SK3 channels, causing a swift influx of Ca^2+^ that triggers PAD4 activation [111]. PAD4 then cooperates with MPO and NE to promote chromatin decondensation. The decondensed chromatin associates with histones and granular proteins, such as MPO and NE, and is packaged into vesicles for extracellular release [112]. This NADPH oxidase–independent mechanism allows viable neutrophils to continue performing functions like chemotaxis and phagocytosis, even after NET extrusion, retaining their capacity to migrate toward and engulf pathogens [113].

NETs can include mitochondrial DNA (mtDNA) [114], and both mitochondria and mtDNA have been implicated in vital NETosis [115]. Despite these insights, the detailed mitochondrial signals and downstream effectors driving this pathway remain to be defined.

Efficient removal of NETs is essential to prevent persistent inflammation. Extracellular DNAases—principally DNase I from the DNase I family (DNase I, DNase1L1, DNase1L2, DNase1L3)—rapidly cleave NET scaffolds [116]. Impaired DNase I activity or the presence of anti-NET antibodies that block its access leads to NET persistence, a feature linked to autoimmune conditions such as systemic lupus erythematosus (SLE) [116,117]. Reduced DNase1L3 expression further compromises NET clearance [118]. In addition to DNase I/II, 3′–5′ exonucleases TREX1 and TREX2 participate in NET turnover [119]. Professional phagocytes augment this process: dendritic cells (DCs) employ DNase1L3 to digest extracellular NET fragments, while macrophages internalize and degrade NETs via lysosomal TREX1 [119]—actions enhanced by macrophage-derived DNase I and C1q-mediated opsonization [120]. Moreover, pro-inflammatory (M1-like) macrophages exhibit superior NET clearance through increased macropinocytosis compared to alternatively activated subsets [121].

## 4. Pathological Effects of NETs in IBD

**Figure 2:**
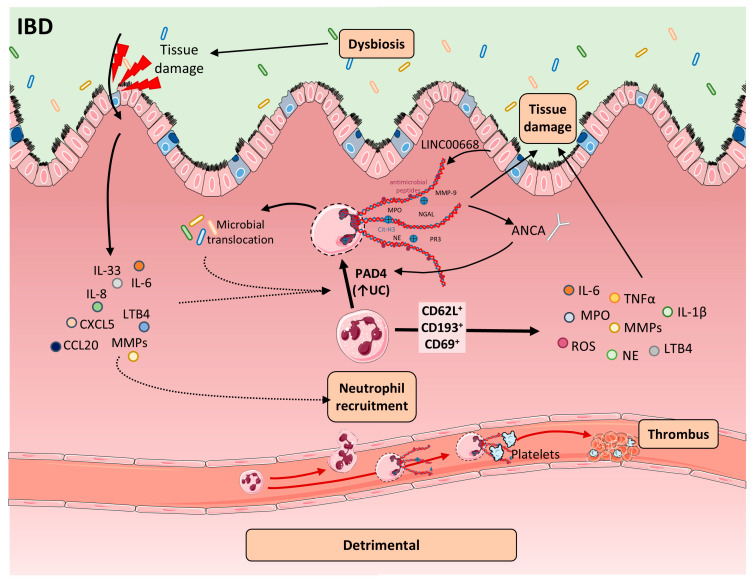
Detrimental role of neutrophils and NETs in IBD. In IBD, inflamed epithelial cells secrete chemotactic factors that orquestate neutrophils recruitment: cytokines (IL-8, IL-6, IL-33); chemokines (CXCL5, CCL20); lipid mediators (LTB4); as well as matrix metalloproteinases (MMPs). Neutrophil hyperactivation and NET production (a process dependent on PAD4, elevated in UC patients) lead to epithelial injury through the overproduction of ROS, cytotoxic granule components (NE, MPO), pro-inflammatory cytokines (TNFa, IL-1b, IL-6) and proteolytic enzymes (MMPs), which disrupt the intestinal mucosal barrier, enhance granulocyte recruitment and NET formation, amplifying the inflammatory cascade. These neutrophils from inflamed tissues exhibit upregulated CD62L, CD193, and CD69. Moreover, NETs release promotes ANCA formation, which promotes neutrophil clustering and NET formation. Also, intestinal epithelial cells from inflamed tissue promote NETs formation through LINC00668. Additionally, the fibrous network of NETs serves as a scaffold for platelet adhesion, erythrocyte entrapment, fibrinogen binding, and recruitment of coagulation factors promoting thrombosis in damaged vessels. Part of the figure was generated by using pictures from Servier Medical Art.

Although originally described in infectious diseases, NETs have been increasingly implicated in various non-infectious conditions, including autoimmune diseases [122], cancer [123,124], diabetes [125,126], organ failure [127], and inflammatory conditions affecting the lungs [128,129] or the cardiovascular system [130,131]. Their potential role in the pathogenesis of IBD has attracted increasing attention in recent years Figure 2.

### 4.1. Increased NET Formation in the Inflamed Intestine

An imbalance favoring NET accumulation has been linked to diverse pathologies—including cystic fibrosis, atherosclerosis, SLE, COVID-19, rheumatoid arthritis (RA), vasculitis, IBD, and cancer—where NETs drive cytokine release, autoantibody formation, thrombosis, and tumor cell dissemination [81,97,132,133].

In IBD, NETs have been detected in inflamed mucosa of both UC and CD patients, correlating with disease activity [49,134,135,136,137], and in the intestine of mouse models of colitis [138]. The cytokine-rich, oxidative milieu of IBD—marked by high levels of TNF-α, IL-1β, and IL-6—and increased microbial translocation provide potent danger-associated molecular pattern (DAMP) and pathogen-associated molecular pattern (PAMP) stimuli that promote neutrophil activation and NET release [73]. Concomitantly, NET clearance mechanisms are compromised—via reduced DNase activity and anti-NET autoantibodies—allowing NET persistence and further fueling inflammation [116,139]. Pro-inflammatory cytokines abundant in the UC microenvironment—such as TNF-α, IL-1β, IL-6, and IL-8—further promote NETosis. TNF-α markedly enhances NET formation in neutrophils from UC patients, an effect reversed by infliximab treatment [140]. NETs, in turn, stimulate mononuclear cells from the lamina propria to produce TNF-α and IL-1β, partly via ERK phosphorylation [140]. IL-8 not only drives neutrophil chemotaxis but also directly induces NET formation [141]. Likewise, IL-6, which is upregulated in UC and murine colitis models, acts as a potent NETosis trigger [142]. NETs also activate macrophages, leading to additional cytokine release and further amplification of the inflammatory cascade [143].

Despite these observations, the intracellular signaling pathways connecting these cytokines to NETosis remain incompletely understood. Elevated TNF-α engages neutrophil TNF receptors to activate NF-κB, upregulating genes essential for NET assembly—including histone deacetylases—and amplifying NET production [144]. Consequently, NETs accumulate in the mucosa during flares—levels are significantly higher in active IBD compared to inactive disease or healthy controls—and remain unchanged in non-inflammatory disorders such as irritable bowel syndrome (IBS), highlighting their specificity to inflammatory pathology [145,146]. Exosomal LINC00668 derived from intestinal epithelial cells (IECs)—which is enriched in inflamed tissue—has been shown to facilitate NE translocation to the nucleus, facilitating chromatin decondensation and histone cleavage in colitic mice [145,147], thereby contributing to the process. Autophagy also regulates NET formation, and its inhibition suppresses ROS generation and NET release [148]. Knockdown of autophagy-related genes such as *ATG7* similarly impairs NETosis [149].

In UC, this process is linked to upregulation of the stress-response protein REDD1 in neutrophils, which drives autophagy-dependent NET formation and promotes IL-1β–mediated inflammation [72]. Within the IBD microenvironment, NETs interact dynamically with epithelial cells to drive extracellular matrix (ECM) degradation and remodeling, enhance thrombotic risk, and disturb the microbiota balance [150]. Additional regulators—such as anti-neutrophil cytoplasmic antibodies (ANCA) and IL-1β—modulate NET abundance in IBD, underscoring multifaceted contributions of NETs to mucosal injury and disease progression [151].

### 4.2. Defective NET Clearance in IBD

In IBD, defective NET degradation plays a major role in their accumulation, alongside enhanced formation [145]. Plasma from patients with active UC shows a reduced capacity to degrade NETs, suggesting that clearance mechanisms are impaired [145]. Compared to healthy controls, patients with IBD exhibit significantly decreased DNase I activity in inflamed mucosa, further compromising NET breakdown [139,152]. Importantly, reduced DNase I activity has also been reported in the blood of male patients in clinical remission [153], pointing to a persistent enzymatic deficit. At the same time, ongoing neutrophil infiltration and activation in affected tissues sustain NET release [145], potentially overwhelming already impaired clearance pathways. While naked DNA is generally non-immunogenic, DNA–histone complexes present in NETs are potent autoantigens in several autoimmune diseases [154]. In IBD, elevated antinucleosomal antibody levels are associated with reduced DNase I activity, supporting a self-reinforcing loop between defective NET degradation and autoimmune reactivity [152]. Beyond impaired DNase I activity, the maintenance of NETs in inflamed tissue exacerbates local inflammation and contributes to dysfunction in phagocytic clearance. Among other factors, this may be driven by increased levels of pro-inflammatory cytokines such as IL-6, TNF α, and IL 1β, which are known to not only promote neutrophil activation and NETosis but also impair the ability of macrophages and DCs to efficiently clear the NET remnants via phagocytosis or extracellular degradation pathways [67]. The accumulation of uncleared NETs exacerbates cytokine production, reinforcing a self-sustaining inflammatory circuit that drives the persistent intestinal inflammation characteristic of UC and CD [67]. Moreover, reduced expression of receptors such as MER tyrosine kinase (MERTK) hampers the recognition and engulfment of NETs by phagocytes. MERTK dysfunction contributes to increased endothelial permeability, disrupted cell–cell junctions, and amplified inflammatory signaling, all of which intensify mucosal injury in IBD [144]. Consequently, defective NET clearance perpetuates a self-sustaining cycle of inflammation and tissue damage, complicating disease resolution.

### 4.3. NETs in Promoting the Recruitment of Immune Cells

NETs contain chemokines such as CXCL1, CXCL2, CXCL5, and CXCL8, which facilitate the recruitment of additional neutrophils and attract monocytes, macrophages, NK cells, DCs, and T cells, predominantly via CCL2-mediated signaling [67,155,156]. Neutrophil-derived α-defensins [157] and calprotectin [158] further amplify leukocyte infiltration at inflammatory sites, thereby exacerbating mucosal injury. The resulting cellular accumulation intensifies tissue damage.

### 4.4. ANCA, Autoimmunity, and NET Amplification

PAD4-driven histone citrullination initiates chromatin decondensation and the extrusion of NETs rich in MPO, NE, and PR3 [159]. These NET components become autoantigenic, triggering autoreactive T and B cell responses and the production of ANCAs—a hallmark in UC and implicated in CD autoimmunity [160,161,162,163]. ANCAs bind surface-exposed MPO and PR3 on neutrophils, sustaining their activation and driving a feedforward loop of NET formation. Serum ANCA levels correlate with colonic NET-associated proteins, reinforcing this self-amplifying cycle in the inflamed mucosa [164]. FcRn, the neonatal Fc receptor, regulates ANCA trafficking by selectively binding IgG and albumin at distinct, nonoverlapping sites within acidic endosomes [165], thereby diverting IgG away from lysosomal degradation and substantially prolonging its serum half-life. In the context of IBD, FcRn sustains ANCA persistence by protecting IgG–antigen complexes from degradation, thereby enhancing their stability and circulation time, which fosters persistent neutrophil activation and NET release [166,167]. The prolonged persistence of these autoantibodies and immune complexes exacerbates tissue injury and sustains chronic inflammation in autoimmune disorders [168]. Targeting FcRn has emerged as a promising strategy, as its blockade accelerates ANCA clearance, reduces mucosal NET burden, and alleviates colonic inflammation in UC models [164]. Consistently, baicalein has been shown to prevent UC relapse by downregulating FcRn expression through inhibition of NF-κB (p50/p65), thereby reducing ANCA levels, NET-associated proteins, pro-inflammatory cytokines, and disease severity in murine models [169].

### 4.5. Neutrophil and NET-Mediated Epithelial Barrier Disruption

In IBD, disruption of the intestinal epithelial barrier—causing damage to both IECs and endothelial cells—impairs mucosal healing, while NETs further weaken this barrier, allowing luminal antigens and microbes to penetrate and exacerbate inflammation [170]. In DSS-colitis models, NETs have been detected not only within the lamina propria and epithelium but also floating freely in the lumen [138]. Functional studies indicate that NETs exacerbate permeability by inducing IEC apoptosis [138]; moreover, Caco-2 monolayers exposed to NETs or purified histones exhibit marked reductions in occludin, ZO-1, and E-cadherin levels compared to controls [171]. In particular, NET-associated histones associate with tight junction complexes, destabilize junctional architecture, and trigger epithelial cell death, collectively increasing barrier leakiness [171,172,173]. NETs isolated from UC patients have been shown to damage both IECs and endothelial cells, suggesting a direct cytotoxic role in mucosal injury [174]. This toxicity likely results from neutrophils traversing the epithelial layer and releasing mediators that compromise epithelial cell integrity [175]. Whether NETs also directly injure endothelial cells in UC remains to be determined. In CD patients, NETs degrade extracellular matrix and tight junction proteins, undermining epithelial integrity and promoting paracellular leakage [176]. Furthermore, NET-packed extracellular vesicles deliver PAD4 into IECs, where PAD4 activation destabilizes mitochondrial creatine kinase 1 (CKMT1 i) via autophagy, contributing to fibrotic remodeling and stricture formation in CD [177].

These findings reveal a dual role for NETs in CD: both disrupting barrier function and altering epithelial cell metabolism toward fibrosis. Moreover, the bioactive monosaccharide allulose has been shown to attenuate colitis in mouse models by preserving tight junction integrity and protecting the intestinal mucosal barrier. In parallel, the detrimental impact of NETs on tight junctions and intestinal barrier integrity has been demonstrated in ischemia–reperfusion models, further highlighting their role in epithelial injury [178].

Interestingly, IECs themselves can enhance NET formation via exosomal LINC00668, which facilitates NE translocation to the nucleus and triggers NET release [147]. Targeting this pathway with berberine—an inhibitor of LINC00668—suppresses NETosis and reduces IL-1β secretion by macrophages, pointing to a novel therapeutic avenue in UC [147].

## 5. Neutrophils and NETs Markers of IBD Progression

Neutrophil infiltration strongly correlates with both endoscopic severity and systemic inflammatory markers in IBD. Among these, serum C-reactive protein and fecal biomarkers such as calprotectin and lactoferrin are routinely used to diagnose IBD and monitor disease activity [38,179]. Additional markers have also been proposed, including fecal [180] or serum [181] MPO activity, fecal NE–antiprotease complexes [182], and fecal serine proteases [183]. Moreover, urinary levels of NE and elafin—an NE inhibitor—have significant diagnostic value in differentiating IBD patients from healthy controls [184] and NGAL levels are high and sensitive in determining diagnosis and disease activity in IBD patients [185]. Moreover, high NGAL expression has been detected in inflamed colonic epithelium and neutrophilic granulocytes [186,187], with fecal NGAL levels correlating with endoscopic and histological findings, as well as with calprotectin concentration [188,189]. Notably, calprotectin, lactoferrin, NE, elafin, NGAL and MPO are major components of NETs and neutrophils [179,180,184,185], further underscoring the association between neutrophil activation, NET formation, and mucosal inflammation in UC and CD.

Regarding UC, several studies have shown that NET-associated proteins, including PAD4, NE, MPO, and Cit-H3, are upregulated and colocalized in active UC lesions, with proteomic analyses confirming NET enrichment in inflamed mucosa compared to healthy tissue [72,140,145,190,191,192]. In parallel, circulating NET levels are elevated in UC patients, and neutrophils from these individuals generate significantly more NETs ex vivo than those from healthy controls [145,193].

Cell-free DNAs (cfDNAs) arise from multiple cellular processes, among which apoptosis, necrosis, NETosis, and active release via microvesicles (MVs) or exosomes are the best characterized [194]. As DAMPs, cfDNAs contribute to inflammation and can promote NETosis when released during tissue injury or cellular stress [195]. In experimental colitis models, including DSS-induced inflammation, increased levels of cfDNA are observed alongside enhanced NET formation [196]. Notably, cfDNA concentrations are also elevated in the serum of patients with UC and CD, as well as in murine models of colitis. These elevated levels correlate with disease activity and actively promote NET release, thereby exacerbating endothelial damage [153,197]. In addition, cfDNA-binding molecules such as histones act as ligands for toll-like receptors TLR2 and TLR4. Activation of these receptors induces the production of inflammatory mediators including TNF-α, IL-6, IL-10, and MPO [198]. In UC, both cfDNA and high-mobility group box 1 (HMGB1) function as potent inducers of NET formation [199,200,201]. Dihydromyricetin, a natural flavonoid with strong antioxidant and anti-inflammatory properties, has been shown to attenuate experimental UC by suppressing NET formation. This effect is reflected in reduced cfDNA levels and diminished inflammatory markers, mediated via modulation of the HIF-1α/VEGFA signaling pathway [202]. HMGB1 exerts similar pro-inflammatory effects by engaging TLR4 and CXCR4 [203,204]. Its expression is markedly elevated in inflamed UC mucosa, and neutralization of HMGB1 in DSS-induced colitis models alleviates barrier dysfunction and intestinal inflammation by regulating NET formation and macrophage polarization [205].

Additionally, serum anti-neutrophil cytoplasmic antibodies (ANCAs) promote neutrophil clustering and NET formation in vivo [164]. Clinically, high PR3-ANCA titers correlate with greater disease severity and guide therapeutic decision-making in UC patients [206]. Moreover, PR3-ANCA levels may help distinguish between UC and CD [207], and their diagnostic utility, along with correlations to disease activity and phenotype, has been demonstrated in pediatric IBD cohorts [162]. PAD4 is a central enzyme in NET formation, mediating histone citrullination and chromatin decondensation. Genetic deletion or pharmacological inhibition of PAD4 blocks NET release from neutrophils [208]. PAD4 expression is significantly upregulated in UC mucosa, particularly in actively inflamed areas, and correlates with histological severity [140,190,209]. In murine colitis, PAD4 deficiency results in reduced inflammation, preserved barrier integrity, and improved clinical outcomes [210]. In this sense, PAD4 genetic knockout also prevents NET-induced activation of the cGAS–STING pathway, thereby reducing pro-inflammatory cytokine release and preserving barrier integrity in colitis models [211].

In CD, NETs have been identified as key mediators of disease pathogenesis [66,212]. By entrapping bacteria and fungi, NETs enhance antigen presentation and activate both innate and adaptive immune responses, thereby sustaining a pro-inflammatory environment in the gut [150]. In turn, NETs recruit macrophages and T cells, promoting the release of pro-inflammatory cytokines such as TNF-α and IL-1β, which further amplify tissue damage [140]. NET-driven inflammation also underlies complex perianal fistula formation in CD [213]. In perianal fistulizing CD distinct microbial communities—enriched in NET-positive fistulas and including *Prevotella bivia*, *Streptococcus gordonii*, and *Bacteroides dorei*—correlate with increased neutrophil and monocyte infiltration, alongside activation of immune and wound healing pathways [214]. These NET-rich microenvironments are also associated with reduced tissue infliximab levels and impaired healing outcomes, underscoring the pivotal role of NETs in modulating microbe-host interactions and determining clinical prognosis in perianal fistulas in CD [214]. Following the role of NETs in CD, six NET-associated hub genes have also been identified as potential diagnostic markers for CD [215]. Moreover, NETs can activate the complement cascade, leading to C5a generation, which in turn attracts additional leukocytes and intensifies inflammatory signaling [216].

## 6. Contribution of NETs to ECM Remodeling and Fibrosis in IBD

ECM provides a structural scaffold that preserves tissue architecture and regulates cellular behaviors such as adhesion, migration, proliferation, and differentiation. It also orchestrates tissue development, repair, and homeostasis through dynamic interactions with cells and soluble factors [217]. ECM remodeling plays a pivotal role in IBD pathogenesis, as chronic gut inflammation disrupts the balance between ECM synthesis and degradation, driven by increased activity of proteases such as MMPs, meprins, and NE [218,219]. This imbalance leads to tissue damage and the release of ECM components into circulation. Persistent inflammation also promotes the activation of myofibroblasts by pro-inflammatory and profibrotic mediators, shifting repair processes toward fibrosis due to excessive ECM deposition [220].

In UC, mucosal biopsies display marked reductions in multiple ECM components, reflecting disrupted matrix integrity rather than downregulated gene expression or protein synthesis—evidenced by the appearance of ECM fragments in the tissue [221]. Concomitantly, elevated activities of proteolytic enzymes—including myeloblastin, neutrophil collagenase, MMP-9, and MMP-10—have been detected in the UC mucosa, implicating these proteases in pathological matrix breakdown [222]. The resulting loss of ECM cohesion likely facilitates ulceration and impairs mucosal repair. Given the established role of NETs in releasing neutrophil-derived proteases and promoting microenvironmental inflammation, it is plausible that NET-associated enzymes contribute directly to ECM degradation in UC [223]. Among these, NE contributes to ECM remodeling in IBD by cleaving collagen, laminin, fibronectin, and elastin, both directly and indirectly through MMP activation, thus promoting tissue injury. It also disrupts intestinal barrier integrity by degrading tight junction proteins such as E-cadherin [150,182]. Through these combined actions, NE plays a central role in promoting inflammation and epithelial injury in IBD. In contrast, elafin—a natural NE inhibitor expressed by neutrophils and other cell types—counteracts these effects by preserving barrier integrity and attenuating inflammation, primarily through NF-κB inhibition and cytokine suppression [224]. Notably, urinary levels of NE, elafin, and particularly the NE/elafin ratio demonstrate significant diagnostic potential for distinguishing IBD patients from healthy individuals [184]. NGAL, released by activated neutrophils, acts as a pro-inflammatory factor and chemoattractant [218,225]. By forming a complex with MMP-9, NGAL stabilizes the enzyme, prolonging its ECM-degrading activity and preventing its inactivation by TIMP-1. The NF-κB pathway, activated by cytokines like TNF-α and IL-1, further enhances NGAL and MMP-9 expression, reinforcing the connection between inflammation and ECM remodeling [218,226]. The diagnostic utility of some of these ECM-related markers in serum such as laminin, fibronectin, and NGAL has also been reported for distinguishing UC from CD and for monitoring disease activity [227].

ECM remodeling also plays a central role in the development of intestinal fibrosis, one of the most common long-term complications in IBD. In CD, luminal narrowing due to strictures or stenosis is common and often progresses to bowel obstruction [228]. Although typically linked to the transmural inflammation of CD, fibrosis with obstruction can also occur in UC, where inflammation remains confined to the mucosa and submucosa [229]. Strictures result from excessive and disorganized ECM deposition, mainly in the submucosa and sometimes extending into the muscularis propria [230]. While traditionally considered a passive consequence of chronic inflammation, ECM is now recognized as an active participant in fibrogenesis through its influence on immune signaling and cell behavior [231]. In the submucosa, PGP—a matrikine produced via MMP-8/9–mediated collagen degradation—is elevated in IBD tissues and drives neutrophil recruitment [232]. Moreover, collagen-derived biomarkers such as PRO-C6 and C4M not only differentiate stenosing from luminal CD but also correlate with histological fibrosis and neutrophil presence, highlighting the role of neutrophils in ECM remodeling [233]. Neutrophil–fibroblast interactions are emerging as key drivers of chronic inflammation and ECM remodeling in IBD [129,234]. Fibroblasts, the main mesenchymal cell population within the intestinal stroma, play a pivotal role in maintaining ECM architecture and responding to inflammatory stimuli. Under pathological conditions, they secrete chemokines such as CXCL1, CXCL8, and CXCL12 that attract neutrophils. These, in turn, release NETs, elastase, and pro-inflammatory cytokines, which further modulate fibroblast activity and promote ECM dysregulation [235].

In CD, this feedback loop becomes particularly relevant. Neutrophils exhibit a profibrotic phenotype in an environment rich in NETs, enhancing collagen production by intestinal fibroblasts through IFN-α–mediated pathways. Fibroblasts respond by releasing IL-8, which sustains neutrophil infiltration and perpetuates inflammation and fibrosis [236]. Additionally, increased IL-17 expression in CD stimulates fibroblasts to upregulate NFKBIZ and CXCL1, promoting neutrophil chemotaxis and intensifying local inflammation [237]. NETs have been shown to co-localize with activated fibroblasts in ulcerated intestinal regions, where they induce the expression of profibrotic genes and activate innate immune signaling cascades, particularly via TLR2/NF-κB. This signaling enhances fibroblast proliferation and ECM synthesis [238].

Gene expression analyses in IBD tissue have revealed that fibroblasts in ulcerated areas acquire a neutrophil-recruiting phenotype, dependent primarily on IL-1 receptor signaling. Moreover, in patient’s refractory to conventional therapies, this neutrophil–fibroblast signature is markedly upregulated, suggesting that disrupting their interaction could be a therapeutic avenue [239].

Further research has identified a fibroblast subpopulation expressing NRG1 and IL1R1, which becomes expanded in inflamed IBD mucosa. Upon epithelial damage, neutrophil-derived IL-1β suppresses reparative NRG1 secretion from these fibroblasts, impairing epithelial regeneration and aggravating inflammation [240].

Therefore, in IBD, ECM remodeling similarly emerges as a hallmark of pathogenesis, reflecting a synergistic interplay between neutrophils, NET formation, and matrix disruption.

## 7. Prothrombotic Effects of NETs in IBD

Patients with IBD exhibit a two-three-fold higher incidence of thromboembolic complications compared to the general population [241]. Persistent intestinal inflammation in UC drives neutrophil activation and NET release, creating a prothrombotic milieu. The fibrous network of NETs serves as a scaffold for platelet adhesion, erythrocyte entrapment, fibrinogen binding, and recruitment of coagulation factors [242]. NETs also directly enhance platelet procoagulant function by presenting phosphatidylserine on their surfaces and by shedding platelet-derived microparticles, both of which amplify thrombin generation [243]. In active UC, elevated platelet microparticles production and increased platelet phosphatidylserine exposure correlate with heightened coagulation potential, thereby raising the risk of venous thromboembolism and cardiovascular events [73,244].

Clinical and experimental data confirm NET-driven thrombosis in UC. The incubation of healthy platelets with NETs from active UC patients increases their procoagulant activity by 32% and fibrin formation capacity by 42% [145]. NET-induced platelet activation involves TLR2 and TLR4 signaling [145], and time-dependent phosphatidylserine exposure on endothelial cells accelerates thrombin and factor Xa complex formation. In DSS-induced colitis models, NET accumulation in vena cava thrombi is linked to inflammatory exosomes released by IECs [147]. The precise mechanisms by which IEC-derived exosomes modulate NET-associated thrombosis in UC warrant further study. Therefore, NET deposition in colonic tissue and circulation is associated with an increased risk of venous thromboembolism during active IBD [145], although NET-mediated immunothrombosis has been shown to reduce bleeding in murine UC models [209].

Therapeutically, DNase administration offers dual benefits in UC thromboinflammation: it degrades NET scaffolds to reduce macrophage-derived prothrombotic cytokines (IL-1β, TNF-α, IL-6) [245,246,247], and it directly inhibits platelet activation, thereby lowering thrombotic risk [248]. These findings highlight DNase as a promising adjunct in managing UC-associated coagulopathy.

## 8. Microbiota and NET Interactions

The gut microbiome—a complex ecosystem of bacteria, archaea, fungi, and viruses—supports host nutrition, mucosal defense, and immune homeostasis [249]. In IBD, high-throughput sequencing has revealed marked shifts in this community, termed dysbiosis, which correlate with disease onset and severity [250]. A consistent finding is reduced bacterial diversity, including loss of Firmicutes and depletion of butyrate-producing taxa such as *Faecalibacterium prausnitzii*—a member of *Clostridium* cluster IV whose decline associates with CD relapse risk [251,252]. Conversely, expansions of Proteobacteria and Bacteroidetes—and in CD, adhesive-invasive *Escherichia coli*—have been reported, contributing to increased permeability and mucosal inflammation [253,254]. These compositional changes disrupt microbial metabolism. In this context, biologic treatment in CD patients has been found to induce shifts in the gut microbiota, with increased Firmicutes and decreased Bacteroidetes, as well as a gradual improvement in the Firmicutes/Bacteroidetes ratio after treatment, correlating with disease activity [255]. Overgrowth of sulfate-reducing genera like *Desulfovibrio* generates toxic sulfide species that injure epithelial cells and trigger mucosal inflammation [256,257,258]. Simultaneously, loss of short-chain fatty acids (SCFAs)—notably butyrate—impairs regulatory pathways: butyrate dampens neutrophil release of pro-inflammatory cytokines and chemokines (CCL3, CCL4, CXCL1, IL-8), limits NET formation, and modulates neutrophil gene expression in pathways of leukocyte activation, innate immunity, and oxidative stress [254,259,260].

Therapeutically, butyrate supplementation in DSS-colitis models reduces weight loss, preserves colon length, and lowers histology scores by curbing neutrophil infiltration, reducing inflammatory mediators, and suppressing NETosis. These effects enhance Treg differentiation and epithelial repair, underscoring the role of butyrate in restoring mucosal equilibrium [261]. Together, these data illustrate how microbial community shifts and altered metabolite output synergistically drive IBD pathogenesis by perturbing epithelial integrity, immune regulation, and neutrophil function.

## 9. Protective Roles of Neutrophils and NETs in IBD

**Figure 3:**
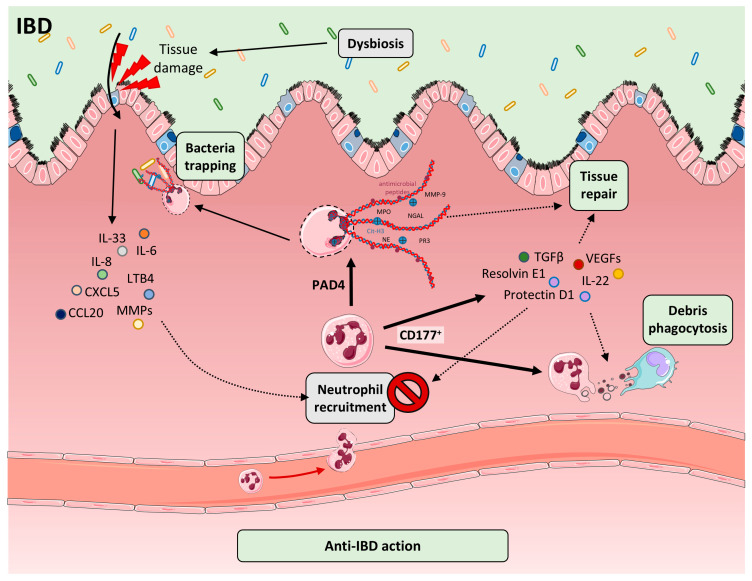
Protective role of neutrophils and NETs in IBD. Neutrophils play a central role in innate immunity in the gut. They mediate antimicrobial activity through phagocytosis, NETosis and degranulation, ultimately leading to pathogen clearance. CD177^+^ neutrophils are proposed to confer protection in IBD. They exhibit enhanced microbial killing by ROS and NETs production, and produce reparative mediators (IL-22, TGFβ, VEGF, protectin D1, resolvin E1), which control further neutrophil influx and promote macrophage clearance of apoptotic neutrophils. Part of the figure was generated by using pictures from Servier Medical Art.

NETs have been implicated in modulating inflammatory responses beneficially across various pathological settings Figure 3. In RA, they have been reported to suppress IL-6 production while enhancing IL-10 secretion in macrophages stimulated with LPS [262]. In the context of gout, NETs facilitate inflammation resolution by degrading cytokines and chemokines through the action of serine proteases; notably, impaired NET formation enhances the pro-inflammatory activation of neutrophils by monosodium urate crystals, the key pathogenic factor in this disease [263]. Proteolytic enzymes embedded within NETs have been shown to modulate cytokine activity effectively in both in vivo and in vitro settings, and are believed to contribute to the regulation of a broad spectrum of cytokines implicated in IBD [264]. Moreover, circulating NETs may participate in the clearance of damage-associated molecular patterns, including translocated bacterial constituents [265,266,267], a function that has been validated in murine models of sepsis [268].

Although neutrophils are commonly associated with tissue injury, the absence of neutrophils in experimental colitis models worsens disease outcomes, highlighting their essential role in mucosal defense and repair [46,269,270]. Beyond pathogen clearance and debris removal, neutrophils facilitate epithelial regeneration and resolution of inflammation [49,59,271]. In response to IBD-associated inflammatory cues, neutrophils exhibit elevated expression of CD177, which facilitates their migratory capacity. CD177^+^ neutrophils exert both bactericidal activity and barrier-protective effects at sites of mucosal inflammation in IBD, while also modulating the release of inflammatory mediators that are strongly associated with disease severity in affected patients. Thus, CD177^+^ neutrophils appear to play a dual role in the pathophysiology of IBD [59]. This specialized subset—CD177^+^ neutrophils—exhibits enhanced microbial killing through ROS generation, AMP release, and NETosis, while limiting pro-inflammatory cytokine output (IL-6, IL-17A, IFNγ) and producing reparative mediators (IL-22, TGFβ), thus supporting tissue healing in IBD [59]. These cells also secrete VEGF and pro-resolving lipids (protectin D1, resolvin E1), which control further neutrophil influx and promote macrophage clearance of apoptotic neutrophils in murine models [271]. CD177^+^ neutrophils also play a crucial role in regulating the BMP signaling pathway in IBD [272]. CD177 expression on neutrophils has been associated with the progression and chronicity of inflammatory conditions [273]. In patients with IBD, transcriptomic profiling of whole blood revealed *CD177* as the most differentially expressed gene, with levels correlating positively with endoscopic activity, suggesting its potential as a biomarker of intestinal inflammation [274].

Other neutrophil subsets restrain inflammation: CXCR4^hi^ neutrophils contribute to angiogenesis and extracellular matrix remodeling in the intestine through the release of MMP-9 [275,276]. In parallel, elevated MCPIP-1 expression in neutrophils from IBD patients has been shown to restrict their migratory capacity and attenuate pro-inflammatory responses [277]. Additionally, neutrophils can disengage from clusters autonomously in both in vivo and in vitro settings, promoting tissue surveillance and limiting excessive accumulation at inflammatory sites [278]. Neutrophils also protect against intestinal inflammation and colitis-associated colorectal cancer by modulating the microbiota and promoting an IL-22–dependent tissue repair pathway [279]. Deficiency of S100a10, a member of the S100 protein family, in neutrophils aggravates experimental colitis [280]. Additionally, ferulic acid-mediated attenuation of colitis is neutrophil-dependent and primarily driven by the suppression of NET formation [281].

Contrary to their pro-inflammatory reputation, NETs also exert anti-inflammatory and barrier-protective effects. In severe sepsis, NETs sequester circulating bacteria, preventing systemic spread [268]. During gout flares, NET-bound serine proteases degrade inflammatory cytokines and chemokines, promoting resolution; impairment of NETosis in this setting leads to uncontrolled mediator release [263]. Similar NET-mediated dampening of inflammation has been observed in periodontitis [282] and RA, where NETs suppress IL-6 and induce IL-10 secretion from macrophages [262]. In necrotizing enterocolitis, NET formation has been shown to reduce microbial translocation and systemic inflammation, whereas NET deficiency leads to increased bacteremia and mortality in murine models [283]. TREM-1 activation enhances NET and IL-22 production by CD177^+^ neutrophils, improving barrier function and pathogen clearance in IBD models [271]. Moreover, PAD4 deficiency worsens DSS-induced colitis and increases rectal bleeding, as PAD4-dependent NETs contribute to immune thrombus formation, limiting hemorrhage in UC [209]. These findings underscore the dualistic nature of neutrophils and NETs: while excessive NETosis can drive pathology, both neutrophil subsets and NET structures provide indispensable anti-inflammatory, reparative, and antimicrobial functions in the gut. Therapeutic modulation of NETs in UC must therefore strike a balance between mitigating harmful inflammation and preserving these critical protective roles.

## 10. Therapeutic Modulation of Neutrophils and NETs in IBD

**Figure 4:**
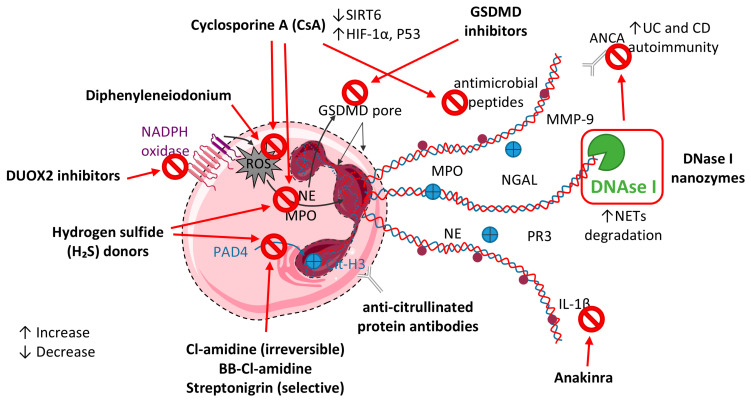
Therapeutic strategies modulating Neutrophils and NETs in IBD. Therapeutic strategies aimed at restoring NET homeostasis while preserving antimicrobial defense include: inhibitors of PAD4 (e.g., Cl-amidine, BB-Cl-amidine, streptonigrin) to block histone citrullination; donors of hydrogen sulfide (H_2_S) to downregulate PAD4, citrullinated histone H3 (Cit-H3), and MPO expression; cyclosporine A (CsA) to suppress neutrophil activation and ROS/MPO release while upregulating HIF-1α/P53 via SIRT6 inhibition; inhibitors of DUOX2 and diphenyleneiodonium to reduce NADPH oxidase-derived ROS; exogenous DNase I (including nanozyme formulations) and anti-citrullinated protein antibodies to enhance NET clearance; gasdermin D (GSDMD) inhibitors to prevent pore formation; and IL-1β blockade (e.g., anakinra) to interrupt pro-NET inflammatory signaling. Part of the figure was generated by using pictures from Servier Medical Art.

Dysregulated neutrophil activity and excessive NET formation play central roles in IBD pathogenesis, positioning both as promising therapeutic targets. Precision strategies must focus on either suppressing NET release or enhancing NET clearance rather than on broad immunosuppression. Natural products and novel small molecules targeting neutrophil activation and NET production are under investigation, reflecting a shift toward personalized IBD management. Nonetheless, comprehensive clinical trials are required to establish the safety, optimal dosing, and long-term efficacy of these innovative interventions [284]; see Figure 4.

### 10.1. PAD4 Inhibition Strategies

Given its pivotal function in NET formation, PAD4 represents an attractive therapeutic target in IBD. Inhibitors such as Cl-amidine, BB-Cl-amidine, and streptonigrin block histone citrullination and chromatin decondensation by PAD4 [285]. Clinically, colonic PAD4 mRNA levels correlate with disease activity and cytokine production in UC and with disease severity and pro-inflammatory responses in CD [286], reinforcing PAD4 inhibition as a promising approach across IBD subtypes. Cl-amidine, an irreversible PAD4 inhibitor, has demonstrated strong efficacy in TNBS-induced colitis by reducing NET formation, lowering colonic PAD4 and Cit-H3 levels, and improving clinical disease scores [287]. Cl-amidine also decreases pro-inflammatory cytokines TNF-α, IL-1β, and IL-6 while increasing IL-10 production [286,287] and exerts antioxidant effects that limit leukocyte activation and protect epithelial DNA integrity [288]. Similarly, streptonigrin, a selective PAD4 inhibitor, has been reported to downregulate pro-inflammatory cytokines and NET-associated proteins, effectively mitigating colonic inflammation [140]. Additionally, recent evidence suggests that hydrogen sulfide (H_2_S) donors may exert protective effects by inhibiting PAD4, Cit-H3, and MPO expression, and by suppressing NF-κB and HMGB1 signaling pathways in TNBS-induced colitis models [289]. Importantly, NETs have been shown to initiate fibroblast activation and collagen deposition via the TLR2/NF-κB pathway in CD, while neutrophil-specific PAD4 deletion attenuates intestinal fibrosis in chronic DSS colitis, supporting PAD4 inhibition as a dual anti-inflammatory and anti-fibrotic strategy in IBD [238].

While preclinical outcomes are encouraging, the clinical translation of PAD4 inhibitors is limited by the paucity of long-term efficacy and safety data in humans. Further clinical investigation is essential to validate their therapeutic potential in IBD.

### 10.2. Targeting NADPH Oxidase and ROS Pathways

Suppressing key neutrophil effectors such as NE and ROS has emerged as a promising strategy to limit NET formation and mitigate inflammation in IBD [290,291]. Cyclosporine A (CsA), an immunosuppressive agent widely used in the management of steroid-refractory acute severe ulcerative colitis (ASUC), exerts immunomodulatory effects not only by inhibiting IL-2 secretion from T cells [292,293], but also by impairing DCs migration [294,295]. Notably, CsA inhibits several neutrophil-mediated processes, including ROS production and NET formation [296], and significantly reduces ROS levels in isolated human neutrophils [292,297]. In ASUC patients, CsA has been shown to suppress neutrophil apoptosis and migration, as well as the release of ROS, MPO, and antimicrobial peptides [298]. At the mechanistic level, CsA downregulates SIRT6, leading to the upregulation of HIF-1α and metabolic reprogramming toward glycolysis and the tricarboxylic acid cycle, which ultimately limits neutrophil overactivation and attenuates mucosal inflammation. Furthermore, recent findings indicate that CsA activates p53, thereby suppressing the activity of glucose-6-phosphate dehydrogenase (G6PD)—a rate-limiting enzyme of the pentose phosphate pathway (PPP)—resulting in decreased ROS generation and reduced NET formation in colitis [193].

In parallel, transcriptional reprogramming of neutrophils has been observed in murine models of colitis and infection, as well as in idiopathic IBD and chronic granulomatous disease-associated IBD. This reprogramming leads to the emergence of neutrophil subsets with high DUOX2 expression, a non-NOX NADPH oxidase [35]. Importantly, DUOX2 upregulation enhances intracellular ROS generation, which not only amplifies chemokine and cytokine release but also promotes NET formation. These context-dependent DUOX2^high^ neutrophils sustain a pro-inflammatory, NET-rich microenvironment that exacerbates mucosal damage and hinders resolution. Accordingly, selective inhibition of DUOX2 may dampen NET production and represents a promising therapeutic avenue to mitigate ROS- and NET-driven inflammation in IBD [35].

Finally, direct inhibition of NADPH oxidase-derived ROS with agents such as diphenyleneiodonium has shown efficacy in experimental models [299]. However, their clinical application remains limited due to adverse effects on neutrophil viability and morphology, which underscore the need for more selective and safer therapeutic alternatives in targeting ROS-driven inflammation in IBD.

### 10.3. Targeting DNase I

DNase I has demonstrated potential as a therapeutic agent in IBD through its capacity to degrade extracellular DNA scaffolds within NETs [138,145,212]. In murine models of DSS-induced colitis, treatment with DNase I resulted in improved stool consistency and reductions in fecal occult blood and rectal bleeding [138]. These clinical parameters were accompanied by enhanced expression of epithelial tight junction proteins such as occludin and ZO-1, indicating restoration of intestinal barrier integrity. In TNBS-induced colitis, enzymatic dismantling of NETs by DNase I further mitigated mucosal damage, downregulated pro-inflammatory cytokine levels, and attenuated tissue inflammation [138]. In animal models, administration of DNase I to degrade NET scaffolds and neutralizing antibodies against NET components effectively attenuates colonic inflammation [212]. Inhibition of upstream mediators—such as complement receptor 3 (CR3) or PDGFRα—also reduces inflammatory and fibrotic responses by preventing NETosis [300]. Complementing these approaches, therapies that boost endogenous DNase I activity or promote phagocytic removal of NET remnants can accelerate inflammation resolution and restore mucosal integrity.

Despite its potential, DNase I suffers from low stability and rapid clearance, limiting its therapeutic application. Recent development of polymeric nanozymes has overcome these issues by extending DNase I activity in the colon with minimal toxicity. In IBD animal models, these DNase I nanozymes not only alleviated key pathological features but also significantly reduced neutrophil infiltration and NETosis compared with free DNase I or mesalamine [212]. The efficacy of DNase I is further constrained by its inability to degrade all NET components, allowing cytotoxic constituents such as histones and proteases to persist. Co-targeting HMGB1 alongside DNase I enhances therapeutic benefit in murine colitis, yielding greater reductions in inflammation than either intervention alone [205]. Therefore, despite its promise, DNase I requires cautious application and case-specific evaluation.

### 10.4. Antibodies and Immune-Modulatory Agents

Certain antibodies, including anti-citrullinated protein antibodies, have been demonstrated to inhibit NET release and may promote their clearance by macrophages [301]. Concurrently, gasdermin D inhibitors exhibit potential to selectively suppress NET formation while maintaining critical neutrophil functions such as phagocytosis [302]. Moreover, blockade of interleukin-1β (IL-1β) signaling, notably through agents like Anakinra, has emerged as a promising approach for NET-targeted therapies. Anakinra is currently being evaluated in a Phase II clinical trial for acute severe UC [303]. Supporting this therapeutic avenue, IL-1 signaling inhibition has yielded positive outcomes in individual UC cases [304], while IL-1-driven stromal-neutrophil interactions characterize a subset of IBD patients refractory to conventional treatments [239].

In conclusion, while a range of biologic agents and pharmacological compounds have demonstrated the capacity to inhibit NET formation, the molecular pathways underlying these effects remain to be fully elucidated. Importantly, the broader physiological repercussions of NET suppression—particularly regarding neutrophil function and innate immune competence—warrant careful consideration. A comprehensive understanding of the dynamic interplay between NET induction, regulation, and clearance is imperative for the development of targeted interventions that neutralize the pathological effects of NETs while preserving essential host defense mechanisms.

## 11. Conclusions and Future Perspectives

Neutrophils are key players in IBD pathophysiology due to their ability to secrete pro-inflammatory cytokines, generate ROS, interact with the microbiota, disrupt epithelial integrity, and form NETs. Among these functions, NET formation has gained particular attention, given its complex and sometimes contradictory roles in the disease. While NETs can contribute to the clearance of pathogens and containment of infection, their excessive or dysregulated production has been linked to tissue damage, epithelial barrier dysfunction, dysbiosis, and thrombotic complications—events that are all relevant in the intestinal context of IBD.

A critical question is whether NETs exert a more dominant role in promoting damage or supporting tissue healing. Evidence suggests that they are more frequently associated with epithelial injury, although some findings indicate that overly suppressing NET formation may impair wound healing. This highlights the need for refined strategies to modulate neutrophil responses, particularly NET release, in a way that minimizes collateral damage without compromising essential repair and defense mechanisms.

Another unresolved issue is whether neutrophils—or specific aspects of their activity such as NET formation—can be effectively targeted therapeutically. To do so, a more precise understanding of the signaling pathways and molecular mechanisms that control NET generation and clearance in IBD is essential. Moreover, the possibility of selectively modulating these processes without broadly inhibiting neutrophil function remains a major therapeutic challenge. Notably, little is known about the degradation and clearance of NETs in inflamed intestinal tissues, which may be as important as their formation in modulating disease progression.

Future therapies should also consider the systemic implications of modifying neutrophil function. Intervening in such a central arm of the innate immune response could lead to unintended effects, including impaired host defense or delayed mucosal repair. Therefore, therapeutic approaches will likely need to be combinatorial, integrating modulation of neutrophil functions with other strategies to address the multifactorial nature of IBD and improve long-term prognosis.

In parallel, there is growing interest in exploring neutrophils and NETs as biomarkers in IBD. Their presence and characteristics might help monitor disease activity, evaluate response to treatment, and distinguish between disease subtypes or stages, paving the way for more precise and individualized care. However, several limitations remain, such as the lack of standardized, sensitive, and non-invasive methods to detect and quantify NETs in clinical samples. Further research is needed to identify the stimuli that trigger NET formation in different phases and phenotypes of IBD, and to develop robust tools that translate these findings into clinical decision-making.

In summary, understanding and manipulating neutrophil biology—particularly NET formation—offers promising opportunities for therapeutic innovation and personalized medicine in IBD. However, progress will depend on resolving key questions about their dual roles, regulatory mechanisms, clinical detectability, and the consequences of their modulation within complex disease contexts.

## Abbreviations

The following abbreviations are used in this manuscript:

aGMAbs	Anti–granulocyte–macrophage colony-stimulating factor antibodies
ANCA	Anti-neutrophil cytoplasmic antibody
CAPS	Cryopyrin-associated periodic syndromes
CAR	Coxsackie-adenovirus receptor
CCL	CC-motif chemokine ligand
CD	Crohn’s disease
cfDNA	Cell-free DNA
cGAS	Cyclic GMP-AMP synthase
Cit-H3	Citrullinated histone H3
CR3	Complement receptor 3
CRP	C-reactive protein
CsA	Cyclosporine A
CXCL	C-X-C motif chemokine ligand
DDIT4/REDD1	DNA damage-inducible transcript 4/regulated in development and DNA damage response 1
DAMP	Damage-associated molecular pattern
DNase	Deoxyribonuclease
DSS	Dextran sulfate sodium
ECM	Extracellular matrix
FcRn	Neonatal Fc receptor
FMF	Familial Mediterranean fever
GWAS	Genome-wide association studies
GSDMD	Gasdermin D
HIF-1α	Hypoxia-inducible factor-1α
HMGB1	High-mobility group box 1
HXA3	Hepoxilin A3
IBD	Inflammatory Bowel Disease
IEC	Intestinal epithelial cell
IL	Interleukin
ILC	Innate lymphoid cells
INFα	Interferon-alpha
JAML	Junctional adhesion molecule-like protein
LPS	Lipopolysaccharide
LTB4	Leukotriene B4
MAPK	Mitogen-activated protein kinase
MCP-1	Monocyte chemoattractant protein-1
MERTK	MER tyrosine kinase
MPO	Myeloperoxidase
MMP	Matrix metalloproteinase
mtDNA	Mitochondrial DNA
NE	Neutrophil elastase
NET	Neutrophil extracellular trap
NF-κB	Nuclear factor-Κb
NGAL	Neutrophil gelatinase-associated lipocalin
NOD2	Nucleotide-binding oligomerization domain 2
NOX	NADPH oxidase
PAD4	Peptidylarginine deiminase 4
PAMP	Pathogen-associated molecular pattern
PKC	Protein kinase C
PMA	Phorbol 12-myristate 13-acetate
PR3	Proteinase 3
PMP	Platelet-derived microparticle
ROS	Reactive oxygen species
SCFA	Short-chain fatty acid
SIRT6	Sirtuin 6
SLE	Systemic lupus erythematosus
STING	Stimulator of interferon genes
TGFβ	Transforming growth factor-β
TLR	Toll-like receptor
TNF-α	Tumor necrosis factor-alpha
UC	Ulcerative colitis
